# Sitagliptin Prevents Inflammation and Apoptotic Cell Death in the Kidney of Type 2 Diabetic Animals

**DOI:** 10.1155/2014/538737

**Published:** 2014-04-08

**Authors:** Catarina Marques, Cristina Mega, Andreia Gonçalves, Paulo Rodrigues-Santos, Edite Teixeira-Lemos, Frederico Teixeira, Carlos Fontes-Ribeiro, Flávio Reis, Rosa Fernandes

**Affiliations:** ^1^Laboratory of Pharmacology and Experimental Therapeutics, Institute for Biomedical Imaging and Life Sciences (IBILI), Faculty of Medicine, University of Coimbra, Azinhaga de Santa Comba, 3000-548 Coimbra, Portugal; ^2^ESAV, Technologies and Health Study Center, Polytechnic Institute of Viseu, 3504-510 Viseu, Portugal; ^3^Educational Technologies and Health Study Center, Polytechnic Institute of Viseu, 3504-510 Viseu, Portugal; ^4^Institute of Immunology, Faculty of Medicine, University of Coimbra, 3000-548 Coimbra, Portugal; ^5^Immunology and Oncology Laboratory, CNC, 3004-517 Coimbra, Portugal; ^6^Center of Ophthalmology and Vision Sciences, Institute for Biomedical Imaging and Life Sciences (IBILI), Faculty of Medicine, University of Coimbra, 3000-548 Coimbra, Portugal

## Abstract

This study aimed to evaluate the efficacy of sitagliptin, a dipeptidyl peptidase IV (DPP-IV) inhibitor, in preventing the deleterious effects of diabetes on the kidney in an animal model of type 2 diabetes mellitus; the Zucker diabetic fatty (ZDF) rat: 20-week-old rats were treated with sitagliptin (10 mg/kg bw/day) during 6 weeks. Glycaemia and blood HbA_1c_ levels were monitored, as well as kidney function and lesions. Kidney mRNA and/or protein content/distribution of DPP-IV, GLP-1, GLP-1R, TNF-*α*, IL-1*β*, BAX, Bcl-2, and Bid were evaluated by RT-PCR and/or western blotting/immunohistochemistry. Sitagliptin treatment improved glycaemic control, as reflected by the significantly reduced levels of glycaemia and HbA_1c_ (by about 22.5% and 1.2%, resp.) and ameliorated tubulointerstitial and glomerular lesions. Sitagliptin prevented the diabetes-induced increase in DPP-IV levels and the decrease in GLP-1 levels in kidney. Sitagliptin increased colocalization of GLP-1 and GLP-1R in the diabetic kidney. Sitagliptin also decreased IL-1*β* and TNF-*α* levels, as well as, prevented the increase of BAX/Bcl-2 ratio, Bid protein levels, and TUNEL-positive cells which indicates protective effects against inflammation and proapoptotic state in the kidney of diabetic rats, respectively. In conclusion, sitagliptin might have a major role in preventing diabetic nephropathy evolution due to anti-inflammatory and antiapoptotic properties.

## 1. Introduction

Diabetes is associated with long-term dysfunction and failure of various organs and tissues, with progressive development of specific complications, including diabetic nephropathy (DN). DN, a major microvascular complication of both type 1 and type 2 diabetes, occurring in about 20–40% of patients, can result in end-stage renal disease (ESRD), further requiring dialysis or transplantation [1, 2].

DN is characterized by excessive accumulation of extracellular matrix, with thickening of glomerular basement membrane and hypertrophy and/or loss of various cell types of the glomerulus and tubules, which ultimately progress to glomerulosclerosis and tubulointerstitial fibrosis [3]. Apart from the major role played by hyperglycemia, diverse factors contribute to the development of DN, such as glomerular hyperfiltration [4] and activation of several metabolic pathways, namely, activation of protein kinase C [5], nonenzymatic glycosylation [6], acceleration of the polyol pathway [7], hexosamine biosynthetic pathway [8], and oxidative stress [9]. Additionally, accumulating evidence also points to a crucial role of the inflammatory process in the development and progression of DN [10–12]. This inflammatory response is mediated by diverse inflammatory cells, including macrophages, monocytes, and leukocytes, as well as other molecules, such as chemokines, adhesion molecules, and inflammatory cytokines, namely, tumor necrosis factor alpha (TNF-*α*) and interleukin-1*β* (IL-1*β*) [12–14]. As the inflammation persists certain vascular lesions are exacerbated, such as endothelial dysfunction, tissue damage, renal fibrosis, and apoptotic cell death [13, 14]. Apoptosis has been increasingly associated with the development and/or progression of diabetic nephropathy [15, 16]. It has been described that glucose-induced ROS production contributes to apoptosis in podocyte [17], mesangial [18], and tubular cells [19], leading to DN progression. Furthermore, high glucose-mediated oxidative stress in tubular cells has been associated with increased levels of proapoptotic proteins such as BAX [19]. It was also observed that high glucose induces an increased ratio of BAX/Bcl-2, associated with cytochrome-c release from mitochondria in renal mesangial cells [18].

A novel class of oral antidiabetic agents, the dipeptidyl peptidase-IV (DPP-IV) inhibitors (or gliptins), such as sitagliptin, has increasingly gained emphasis in the therapeutic managing of T2DM patients by potentiating the action of incretins [20, 21]. Incretins are peptide hormones that are involved in the physiologic regulation of glucose homeostasis, namely, by glucagon-like peptide-1 (GLP-1) and glucose-dependent insulinotropic polypeptide (GIP). In fact, incretin hormones are secreted from the gastrointestinal tract after food intake, in a nutrient-dependent manner, and stimulate glucose-dependent insulin secretion [22]. However, in T2DM patients the incretin effect is blunted; the “incretin defect,” is accompanied by reduced bioavailability of incretins, in part due to rapid inactivation by DPP-IV [23]. Several clinical trials in patients with T2DM showed that sitagliptin was well tolerated and significantly improves glycaemic levels, by lowering blood glucose and HbA_1c_ levels [24–28]. Moreover, it has been demonstrated in both type 1 and type 2 diabetes animal models that treatment with DPP-IV inhibitors preserves islet function and increases pancreatic insulin content, through an increase in proliferation, neogenesis, and apoptosis resistance of *β*-cells [29].

We and other authors have been exploiting the cytoprotective actions of DPP-IV inhibitors in distinct organs and pathological conditions, including in pancreas, retina, and heart disorders [30–32]. However, until now, few studies have addressed the putative beneficial impact of these agents, including sitagliptin, on DN [33–35]. In addition, the impact of sitagliptin therapy on inflammation and apoptosis underlying DN development remains relatively unexploited. Several experimental studies have shown that different therapeutic strategies prevent the development or ameliorate renal injury in diabetes, suggesting that modulation of the inflammatory and apoptotic processes is a potential therapeutic target to prevent renal injury in animal models of diabetes [36–38]. Therefore, this study aimed to evaluate the efficacy of sitagliptin in preventing the deleterious effects of diabetes on the kidney of* Zucker diabetic fatty* (ZDF) rats, focusing on anti-inflammatory and antiapoptotic properties.

## 2. Materials and Methods

### 2.1. Animal Model

In this study, we used a rodent model of obese type 2 diabetes mellitus (T2DM), the ZDF rats (ZDF,* fa/fa*), which has a mutation in the gene coding the leptin receptor (*fa/fa*), developing obesity, hyperlipidaemia, fasting hyperglycemia, hyperinsulinemia, and insulin resistance [39], which are features of the T2DM occurring in humans [39], including the presence of microvascular complications, such as DN [35, 40, 41]. ZDF rats (ZDF,* fa/fa*) and their littermates (ZL, +/+) were obtained from Charles River Laboratories (Barcelona, Spain). Rats were housed under controlled temperature (23 ± 1°C) and relative humidity (60%), and a 12-h light-12-h dark cycle was maintained. The animals were fed distilled water* ad libitum* and rodent maintenance chow (A-04 Panlab, Barcelona, Spain) containing 15.4% of protein and 2.9% of lipids. All procedures involving animals were performed according to the National and European Communities Council Directives on Animal Care and approved by the Institutional Ethics Committee of the Faculty of Medicine, University of Coimbra, for animal care and use (approval ID: 015-CE-2011).

In this study, diabetic ZDF (*fa/fa*) and nondiabetic ZDF (+/+) rats that are 20 weeks of age were used. ZDF (*fa/fa*) rats were divided into two groups (*n* = 6–8 rats per group) and one of the groups was orally treated during 6 weeks with vehicle (orange juice) or sitagliptin (Januvia, MSD, Portugal) 10 mg/kg/day. Body weight and glycaemia of diabetic (ZDF (*fa/fa*)) and nondiabetic (ZDF (+/+)) rats were monitored at the beginning (20-week-old) and at the end (26-week-old) of the study.

### 2.2. Sample Collection

Before blood sample collection, rats were anaesthetized (2 mg/kg intraperitoneal cocktail of a 2 : 1 50 mg/mL ketamine solution in 2.5% chlorpromazine) and blood from the jugular vein was collected. After that, rats were sacrificed and the kidneys immediately removed and carefully cleaned of adherent fat and connective tissue. The kidneys were embedded in OCT tissue embedding matrix (Thermo Scientific, Waltham, MA, USA) at −50°C, for immunohistochemistry and fluorescence microscopy studies, or frozen in liquid nitrogen and then stored at −80°C, for immunoblot analysis.

### 2.3. Biochemical Data

Blood samples were collected from the jugular vein. Serum nonfasting glucose levels were measured using a glucose oxidase commercial kit (Sigma-Aldrich, St. Louis, Mo, USA) and HbA_1c_ levels were measured in total blood by DCA 2000+ Analyzer (Bayer Diagnostics, Barcelona, Spain), according to the instructions of the manufacturer. Serum creatinine and blood urea nitrogen (BUN) concentrations were evaluated as renal function indexes and serum total cholesterol (Total-c) and triglycerides (TGs) as lipid profile measures, through automatic validated methods and equipment (Hitachi 717 analyser, Roche Diagnostics Inc., MA, USA).

### 2.4. Histopathological Analysis

Haematoxylin and eosin (H&E) staining: samples were fixed in Bock's fixative embedded in paraffin wax and 3 *μ*m thick sections were stained for routine histopathological diagnosis with HE. Periodic Acid of Schiff (PAS) staining: PAS was used to evaluate and confirm the levels of mesangial expansion, thickening of basement membranes and sclerotic parameters. Samples were fixed in neutral formalin 10% and embedded in paraffin wax and 3 *μ*m thick sections were immersed in water and subsequently treated with a 1% aqueous solution of periodic acid and then washed to remove any traces of the periodic acid and finally treated with Schiff's reagent. All samples were examined by light microscopy using a Microscope Zeiss Mod. Axioplan 2. The degree of injury visible by light microscopy was scored in a double blinded fashion by two pathologists. Lesions were evaluated on the total tissue on the slide. Histopathology: glomerular damage was assessed by evaluating mesangial expansion, glomerular basement membrane and capsule of Bowman thickening, nodular sclerosis, glomerulosclerosis, atrophy, and hyalinosis of the vascular pole. Analyzed tubulointerstitial lesions comprised inflammation, presence of hyaline cylinders, tubular basement membrane irregularity, tubular calcification, and the association of interstitial fibrosis and tubular atrophy (IFTA). Glomerular lesions were grades using a semiquantitative scale for each slide ranging from normal (or minimal) to severe (extensive damage) that was assigned to each component. Severity was graded as absent/normal, mild, moderate, and severe. Scoring was defined according to the extension occupied by the lesion (% area of the tubulus): normal: <25%; mild: 25–50%; moderate: 50–75%; severe: >75%. Tubulointerstitial damage was evaluated and graded by the same semiquantitative method, with the exception of IFTA, which was graded as normal if absent and as mild, moderate, and severe if present in <25%, between 25 and 50%, and over 50% of affected area. When using PAS, the rating was set for intensity and extension of staining, ranging from 0 (no staining) to 3 (intense and extensive staining), respecting tissue specificity scoring when adequate.

### 2.5. Western Blotting

Kidney sections were weighted, cut into small pieces, and homogenized by mechanical dissociation using a Potter-Elvehjem, at 4°C, in 5 volumes of RIPA lysis buffer (150 mM NaCl, 50 mM Tris (pH 7.5), 5 mM ethylene glycol tetraacetic acid (EGTA), 1% Triton X-100 (Tx-100), 0.5% sodium deoxycholate (DOC), and 0.1% sodium dodecyl sulfate (SDS), supplemented with 2 mM phenylmethylsulfonyl fluoride (PMSF), 2 mM iodoacetamide (IAD), 30 mM NaF, 1 mM sodium orthovanadate, and 1x protease inhibitor cocktail (Roche, Indianapolis, IN, USA)). After incubation on ice for 1 h, the lysates were sonicated and then centrifuged at 16,000 ×g, for 15 min, at 4°C. After centrifugation, the resulting supernatant fractions were used to determine protein concentration using the bicinchoninic acid assay (Pierce, Rockfor, IL, USA) and then were denatured with Laemmli buffer.

For the western blot analysis, 40 to 80 *μ*g of protein were loaded per lane on SDS-PAGE. Following electrophoresis and transfer to polyvinylidene difluoride membranes (Immobilon-P PVDF transfer membranes 0,45 *μ*m, Millipore, Billerica, MA, USA), the blots were incubated with rabbit polyclonal anti-CD26 and mouse monoclonal anti-GLP-1 antibodies from Abcam (Cambridge, UK), mouse monoclonal anti-Bcl-2 and rabbit polyclonal anti-BAX from Santa Cruz Biotechnology (Santa Cruz, CA, USA), and rabbit polyclonal anti-Bid from Millipore and mouse monoclonal anti-*β*-actin antibody from Sigma-Aldrich. The bands intensity was detected by ECL reagent (Bio-Rad, Hercules, CA, USA) using an imaging system (VersaDoc 4000 MP, Bio-Rad). Densitometric analyses were performed using the ImageJ 1.42n software.

### 2.6. Immunohistochemistry in Kidney Sections and Fluorescence Microscopy

Transverse sections of rat kidneys (6 *μ*m) were fixed with cold acetone for 10 min. The sections were then washed with phosphate-buffered saline (PBS), permeabilized for 30 min with 0.25% Tx-100 in PBS, and blocked for 40 min with 10% normal goat serum or with 5% BSA, prior to incubation overnight at 4°C with primary antibodies rabbit anti-CD26, mouse anti-GLP-1 and rabbit anti-GLP-1R from Abcam, and goat anti-IL-1*β* and rabbit anti-TNF-*α* from R&D Systems (Minneapolis, MN, USA). The sections were rinsed with PBS and then incubated with 4′,6-diamidino-2-phenylindole (DAPI) and secondary fluorescent antibodies for 1 h at room temperature. After washing, samples were imaged using a confocal microscope (LSM 710, Carl Zeiss, Gottingen, Germany).

### 2.7. RT-qPCR Kidney Gene Expression

#### 2.7.1. Total RNA Isolation

The kidney was stored in RNAlater solution (Ambion, Austin, TX, USA). For RNA extraction, 10 mg of tissue was weighted and 450 *μ*L of RLT Lysis Buffer was added; tissue disruption and homogenization for 2 min at 30 Hz was performed using a TissueLyser (Qiagen, Hilden, Germany). Tissue lysates were processed according to the RNeasy Mini Kit protocol (Qiagen). Total RNA was eluted in 50 *μ*L of RNase-free water. In order to quantify the amount of total RNA extracted and to verify RNA integrity (RIN, RNA Integrity Number), samples were analyzed using a 6000 Nano Chip kit, in the Agilent 2100 bioanalyzer (Agilent Technologies, Walbronn, Germany) and the 2100 expert software, following manufacturer's instructions. The isolation yield was from 0.5 to 3 *μ*g; RIN values were 6.0–9.0 and purity (A260/A280) was 1.8–2.0.

#### 2.7.2. Reverse Transcription

RNA was reverse transcribed with SuperScript III First-Strand Synthesis System for RT-PCR (Invitrogen, California, USA). One microgram of total RNA was mixed with a 2 × First-Strand Reaction Mix and a SuperScript III Enzyme Mix (Oligo (dT) plus Random hexamers). Reactions were carried out in a thermocycler Gene Amp PCR System 9600 (Perkin Elmer, Norwalk, CT, USA), 10 min at 25°C, 30 min at 50°C, and 5 min at 85°C. Reaction products were then digested with 1 *μ*L (2 U) RNase H for 20 min at 37°C and, finally, cDNA was eluted to a final volume of 50 *μ*L and stored at −20°C.

#### 2.7.3. Relative Gene Expression Quantification

Gene expression was performed using a 7900 HT Sequence Detection System (Applied Biosystems, Foster City, USA). A normalization step preceded the gene expression quantification, using geNorm Housekeeping Gene Selection kit for* Rattus norvegicus* (Primer Design, Southampton, UK) and geNorm software (Ghent University Hospital, Center for Medical Genetics, Ghent, Belgium) to select optimal housekeeping genes for this study [42]. Real-time PCR reactions used specific QuantiTect Primer Assays (Qiagen) with optimized primers for BAX, Bcl-2, IL-1*β*, and TNF-*α*. Endogenous controls were also used: glyceraldehyde-3-phosphate dehydrogenase, *β*-actin, and topoisomerase I together with a QuantiTect SYBR Green PCR Kit (Qiagen) used according to manufacturer's instructions. RT-qPCR reactions were carried out with 100 ng cDNA sample, primers (50–200 nM), and 1x QuantiTect SYBR Green PCR Master Mix. Nontemplate control reactions were performed for each gene, in order to assure nonspecific amplification. Reactions were performed with the following thermal profile: 10 min. at 95°C plus 40 cycles of 15 seconds at 95°C and 1 min. at 60°C. Real-time PCR results were analyzed with SDS 2.1 software (Applied Biosystems) and quantification used the 2^−ΔΔCt^ method [43]. The results were obtained in CNQR (calibrated normalized relative quantities) and expressed as arbitrary units.

### 2.8. Apoptosis Assay

Apoptotic cell death was detected by terminal deoxynucleotidyl transferase-mediated dUTP nick-end labeling (TUNEL) assay, using the* in situ* Cell Death Detection Kit, Fluorescein (Roche). Briefly, kidney frozen sections (6 *μ*m) were fixed with 1% paraformaldehyde and permeabilized with 1% Tx-100 in 1% sodium citrate (pH 6) for 2 min on ice. Slides were washed in PBS and then incubated for 60 min at 37°C with TUNEL reaction mix. The nuclei specimens counterstained with DAPI were analysed with the confocal microscope.

### 2.9. Statistical Analysis

Data are expressed as mean ± standard errors of the mean (SEM). The comparison of values between groups was performed by using analysis of variance (ANOVA) followed by Bonferroni's* post hoc* test (GraphPad Prism 5.0 software, La Jolla, CA, USA). Values of *P* < 0.05 were considered statistically significant.

## 3. Results

### 3.1. Sitagliptin Prevents the Weight Loss and Decreases Glucose, HbA_1c_, and TGs Blood Levels in the Diabetic Animals

At the beginning of the treatment (20 weeks), no significant differences in the body weight were observed between the groups. At the end of the study, 26 weeks of age, the diabetic animals exhibited a 15.5% reduction in their body weight (*P* < 0.01) when compared with the control nondiabetic rats. The loss of body weight induced by diabetes was partially prevented in the diabetic rats treated with sitagliptin (Table 1). No differences in food and water intake were found between groups throughout the experimental period (data not shown).

At the beginning of the study (20-week-old animals), the blood glucose levels of diabetic ZDF rats (304.60 ± 9.10 mg/dL) were already significantly higher than those of the age-matched controls (92.30 ± 2.50 mg/dL; *P* < 0.001); hyperglycemia was accompanied by a decline in insulin secretion, as previously reported [44]. Hyperglycemia has aggravated between 20 and 26 weeks (425.40 ± 10.30 mg/dL) in the diabetic animals; an effect that was prevented in the diabetic rats under sitagliptin therapy (329.80 ± 24.40 mg/dL; *P* < 0.001), by significantly reducing glycaemia levels at the end of the study (Table 1).

**Figure 1:**
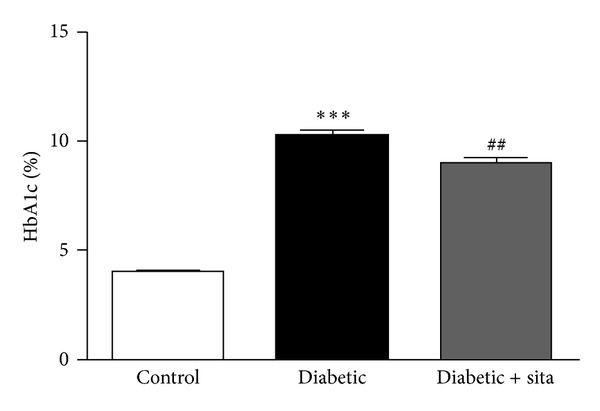
HbA_1c_ levels in control nondiabetic ZDF (+/+) and diabetic ZDF (*fa/fa*) rats nontreated and treated with 10 mg/kg/day sitagliptin for 6 weeks, at 26 weeks of age. Data are expressed as mean ± SEM (*n* ≥ 6 per group); ***P < 0.001 significantly different from control; ^  ##^ *P* < 0.01 significantly different from diabetic.

The HbA_1c_ levels were determined only at the end of the study, in 26-week-old animals (Figure 1). The diabetic group had significantly higher HbA_1c_ levels (10.18 ± 0.29%) than the control group (3.96 ± 0.07%; *P* < 0.001). The diabetic rats under sitagliptin treatment during 6 weeks presented a significantly lower value of HbA_1c_ (8.97 ± 0.21%; *P* < 0.01), with a reduction of 1.2% when compared with nontreated diabetic animals (Figure 1).

The diabetic group presented significantly higher serum contents of Total-c (155.50 ± 3.50 mg/dL, *P* < 0.001) and TGs (374.50 ± 4.95 mg/dL, *P* < 0.05) when compared with the control group (Total-c: 77.50 ± 1.50 mg/dL and TGs: 115.00 ± 11.00 mg/dL), at the baseline (20-week-old animals). After 6 weeks of treatment (Tf: 26 weeks), serum Total-c and TGs levels were maintained higher in the diabetic rats (Total-c: 193.00 ± 9.79 mg/dL, *P* < 0.001; TGs: 400.20 ± 27.00 mg/dL, *P* < 0.001) when compared with the control animals (Total-c: 93.00 ± 2.96 mg/dL; TGs: 154.00 ± 19.14 mg/dL), while significantly reduced serum TGs contents (237.10 ± 22.54 mg/dL, *P* < 0.001) and unchanged Total-c levels (193.10 ± 4.62 mg/dL) were found, when compared with the diabetic vehicle-treated animals.

### 3.2. Sitagliptin Ameliorates Kidney Function and Lesion of Diabetic Animals

The diabetic group presented significantly higher serum contents of BUN (18.15 ± 0.84 mg/dL, *P* < 0.001) and unchanged values of creatinine (0.55 ± 0.06 mg/dL, *P* < 0.05) when compared with the control group (BUN: 14.35 ± 0.47 mg/dL and creatinine: 0.55 ± 0.03 mg/dL), at the baseline (20-week-old animals). After 6 weeks of treatment (Tf: 26 weeks), serum BUN levels (18.03 ± 1.20, *P* < 0.01) were maintained higher and creatinine unchanged (0.54 ± 0.08 mg/dL) in the diabetic rats when compared with the control animals (BUN: 15.05 ± 0.54 mg/dL; creatinine: 0.53 ± 0.03 mg/dL), while a significantly reduced serum BUN concentration (15.16 ± 0.61 mg/dL, *P* < 0.05) and unchanged creatinine contents (0.49 ± 0.04 mg/dL) were found, when compared with the diabetic vehicle-treated animals.

**Figure 2:**
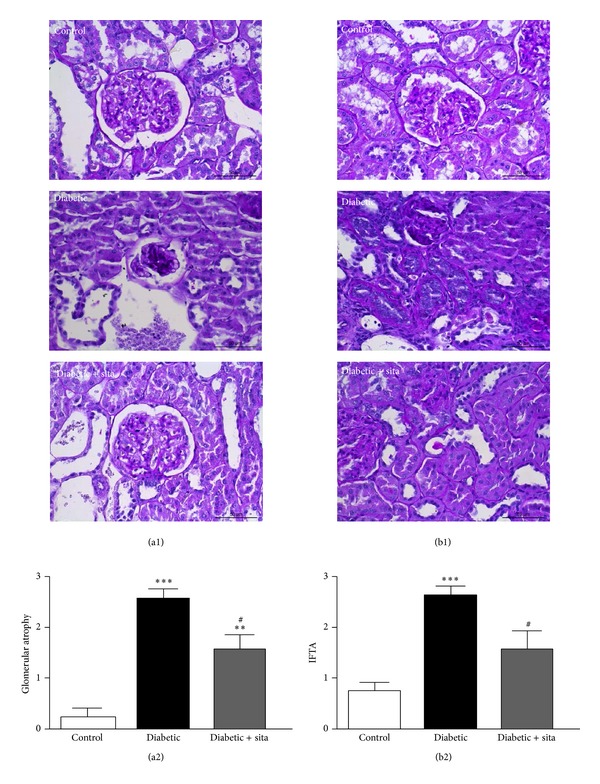
Effects of sitagliptin treatment on renal lesions in the kidney induced by diabetes, such as glomerular atrophy (a) and interstitial fibrosis/tubular atrophy (IFTA) (b) in ZDF (+/+) and ZDF (*fa/fa*) nontreated or treated with sitagliptin. (a1) and (b1) are representative images of renal histology (PAS staining) Data are expressed as mean ± SEM (*n* ≥ 6 per group); **P < 0.01 and ***P < 0.001 significantly different from control; ^#^ *P* < 0.05 significantly different from diabetic.

Concerning kidney lesions, several glomerular and tubulointerstitial lesions were analyzed using HE and PAS staining (Figure 2).* Glomerular lesions: *when aged 20 weeks, the diabetic rats already presented mesangial expansion, nodular sclerosis, glomerulosclerosis, and glomerular atrophy, accompanied by thickening of both glomerular basement membrane and capsule of Bowman. When aged 26 weeks, the lesions were aggravated, showing glomerular basement membrane thickening and glomerular atrophy, when compared with the control animals, accompanied by a significantly more intense expression of mesangial expansion and capsule of Bowman thickening. Glomerulosclerosis was also significantly more obvious in diabetic subjects.

The diabetic rats treated with sitagliptin presented amelioration of kidney glomerular lesions, viewed by reduction of fibrosis and global glomerulosclerosis severity, which is in agreement with the less severe nodular sclerosis, together with reduced hyalinosis of the vascular glomerular pole, mesangial expansion, glomerular atrophy, and glomerular basement membrane thickening, when compared with the diabetic untreated rats. Figure 2(a) shows the significant (*P* < 0.05) amelioration of glomerular atrophy in the diabetic rats treated with sitagliptin when compared with the untreated animals, which presented a significantly increased (*P* < 0.001) grade of glomerular atrophy versus the control group.

*Tubulointerstitial lesions*: when aged 20 weeks, the diabetic rats already presented tubular degeneration, tubular basement membrane irregularity, and IFTA, when compared with the controls animals. The lesions were more profound at 26 weeks, viewed by aggravation of hyaline cylinders, tubular basement membrane irregularity, and IFTA, together with tubular degeneration. Sitagliptin treatment in the diabetic rats prevented the appearance of hyaline cylinders and decreased basement membrane irregularity, tubular degeneration, and IFTA, when compared with the diabetes untreated rats. Figure 2(b) shows the significant (*P* < 0.05) amelioration of IFTA in the diabetic rats treated with sitagliptin when compared with the untreated animals, which presented a significantly increased (*P* < 0.001) IFTA versus the control group.

### 3.3. Sitagliptin Prevents the Upregulation of DPP-IV Content and Modulates the Incretin Axis in the Kidney of Diabetic Animals

**Figure 3:**
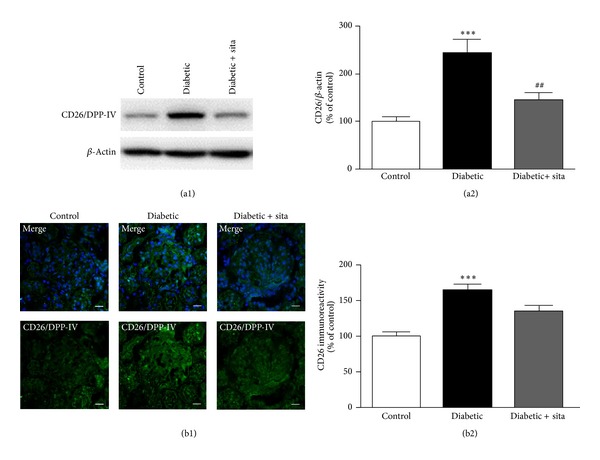
Sitagliptin prevents the upregulation of DPP-IV content in the kidney induced by diabetes. (a) The protein levels of DPP-IV were assessed in total kidney cell lysates by Western Blotting in ZDF (+/+) and ZDF (*fa/fa*) nontreated or treated with sitagliptin. The Western Blots presented are representative of each group of animals. (b) Representative confocal images for each group of animals showing DPP-IV (green) immunoreactivity and nuclear staining with DAPI (blue) in kidney sections (b1), as well as immunoreactivity quantification for DPP-IV (a2). Bars = 20 *μ*m. Data are expressed as percentage of control and represent the mean ± SEM (*n* ≥ 6 per group); ***P < 0.001 significantly different from control; ^##^ *P* < 0.01 significantly different from diabetic.

**Figure 4:**
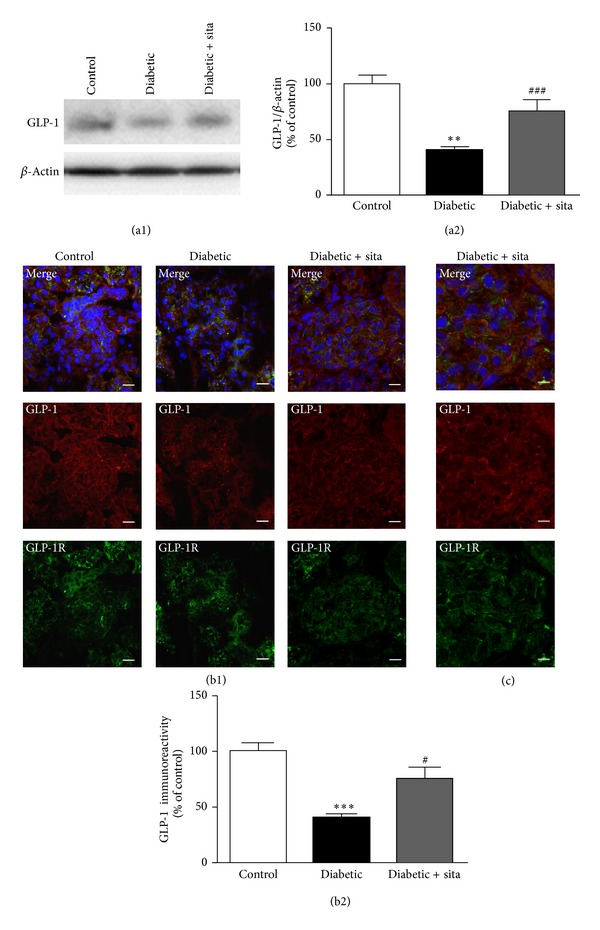
Sitagliptin prevents the downregulation of GLP-1 and GLP-1R content in the kidney induced by diabetes. (a) The protein levels of GLP-1 were assessed in total kidney cell lysates by Western Blotting in ZDF (+/+) and ZDF (*fa/fa*) nontreated or treated with sitagliptin. The Western Blots presented are representative of each group of animals. (b) Representative confocal images for each group of animals showing GLP-1 (red) and GLP-1R (green) immunoreactivity and nuclear staining with DAPI (blue) in kidney sections (b1), as well as immunoreactivity quantification for GLP-1 (b2). Bars = 20 *μ*m. Data are expressed as percentage of control and represent the mean ± SEM (*n* ≥ 6 per group); **P < 0.01 and ***P < 0.001 significantly different from control; ^#^ *P* < 0.05 and ^###^ *P* < 0.001 significantly different from diabetic. (c) Magnification of representative confocal images for the group of ZDF (*fa/fa*) treated with sitagliptin showing colocalization areas of GLP-1 and GLP-1R (yellow) in kidney sections (6 *μ*m). Bars = 10 *μ*m.

To investigate the effect of sitagliptin on DPP-IV, GLP-1, and GLP-1R in the kidney the protein levels and distribution of both proteins were evaluated by* Western Blotting* and immunohistochemistry, respectively (Figures 3 and 4).

Diabetes led to increased DPP-IV levels (244.3 ± 29.1% of control; *P* < 0.001) (Figure 3(a)). Sitagliptin treatment in the diabetic rats prevented the increase in kidney DPP-IV protein levels (146.0 ± 15.7%; *P* < 0.01), compared to diabetic animals without treatment (Figure 3(a)). Immunohistochemistry experiments performed in kidney frozen sections confirmed these results (Figure 3(b)). Diabetes led to a significant increase in DPP-IV glomerular and tubular immunoreactivity. Semiquantitative analysis of glomerular DPP-IV fluorescence intensity revealed significantly lower glomerular DPP-IV levels in diabetic rats treated with sitagliptin (Figure 3(b)).

In diabetic animals, there was a significant decrease in the GLP-1 protein levels (68.2 ± 4.4%; *P* < 0.01), compared to control group (100.0 ± 3.7%). The administration of sitagliptin to diabetic rats increased GLP-1 levels (116.8 ± 15.1%; *P* < 0.001) in diabetic animals relatively to nontreated diabetic rats (Figure 4(a)). By immunohistochemistry, we also observed a decreased immunoreactivity for GLP-1 (from 100.0 to 38.2 ± 3.1%; *P* < 0.001) in the diabetic kidneys. Treatment with sitagliptin increased GLP-1 staining (from 38.2 ± 3.1% to 71.1 ± 10.1%; *P* < 0.05) in diabetic animals (Figure 4(b)). In agreement with these results, GLP-1R immunoreactivity was also decreased in the diabetic kidneys and sitagliptin was also able to increase the GLP-1R staining in the diabetic animals (Figure 4(b)). GLP-1 and GLP-1R costaining analysis revealed that sitagliptin increases the colocalization of the two proteins in the diabetic kidney tissue (Figure 4(c)).

### 3.4. Sitagliptin Decreases the Inflammatory State in the Diabetic Kidney

**Figure 5:**
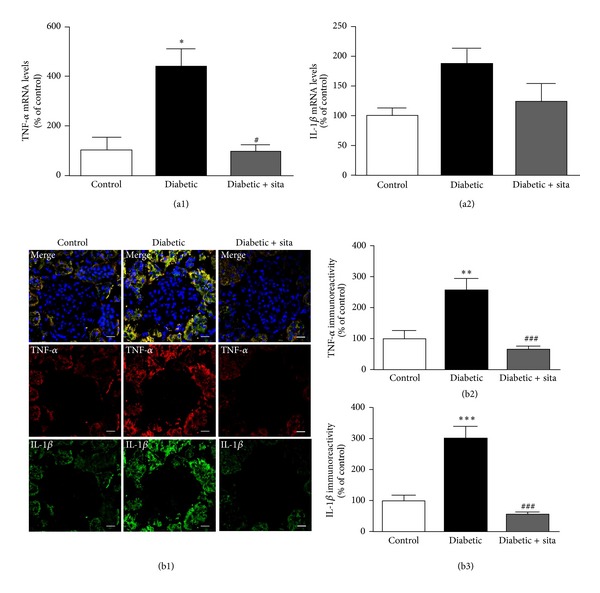
Sitagliptin decreases the proinflammatory cytokines IL-1*β* and TNF-*α* in the diabetic kidney. (a) mRNA expression of mediators of inflammation, TNF-*α* (a1) and IL-1*β* (a2), in the kidney. (b) Representative confocal images showing TNF-*α* immunoreactivity (red), IL-1*β* immunoreactivity (green), and nuclear staining with DAPI (blue) in kidney sections (b1), as well as immunoreactivity quantitation for TNF-*α* (b2) and IL-1*β* (b3). Bars = 20 *μ*m. Data are expressed as percentage of control and represent the mean ± SEM (*n* ≥ 6 per group); *P < 0.05, **P < 0.01, and ***P < 0.001 significantly different from control; ^#^ *P* < 0.05 and ^###^ *P* < 0.001 significantly different from diabetic.

The proinflammatory cytokines IL-1*β* and TNF-*α* are thought to contribute to an inflammatory response in the diabetic kidney. Therefore, their cellular distribution was evaluated in kidney frozen sections by immunohistochemistry and their mRNA levels by RT-qPCR (Figure 5).

The relative expression of TNF-*α* mRNA in the diabetic kidney (437.1 ± 73%; *P* < 0.05) was significantly higher when compared to control animals (100 ± 51%); sitagliptin treatment prevented the effect in the diabetic rats (96.5 ± 24.4%; *P* < 0.05) (Figure 5(a1)). Although no significant differences were observed, the relative expression of IL-1*β* mRNA levels shows a trend to increase in diabetic animals (186.4 ± 25.1%) comparatively to nondiabetic animals (100.0 ± 11.9%); once again, sitagliptin administration presented a trend to reduce IL-1*β* mRNA levels in the diabetic animals (122.9 ± 30.6%) (Figure 5(a2)). Additionally, diabetes markedly increased the immunoreactivity of IL-1*β* and TNF-*α* in cells around the glomeruli that are probably tubular cells and/or recruitment and accumulation of interstitial inflammatory cells; sitagliptin treatment decreased the overexpression of IL-1*β* and TNF-*α* protein levels in the diabetic kidney (Figures 5(b1) and 5(b2)).

### 3.5. Sitagliptin Protects the Diabetic Kidney against Apoptotic Cell Death Induced by Diabetes

It is well established that the ratio between BAX, a proapoptotic protein, and Bcl-2, an antiapoptotic protein, determines the response to a cell death signal, being considered an indicator for the activation of apoptosis [45]. The levels of BAX and Bcl-2 were determined by* Western Blotting*, in total kidney of ZDF (+/+) and ZDF (*fa/fa*) rats, as well as BAX and Bcl-2 mRNA levels. In addition, it has been described that Bid, a proapoptotic protein member of the Bcl-2 family, has an important role in mitochondrial cell death pathway [46]. In this context, we also evaluated Bid protein levels in the kidney by* Western Blotting*.

**Figure 6:**
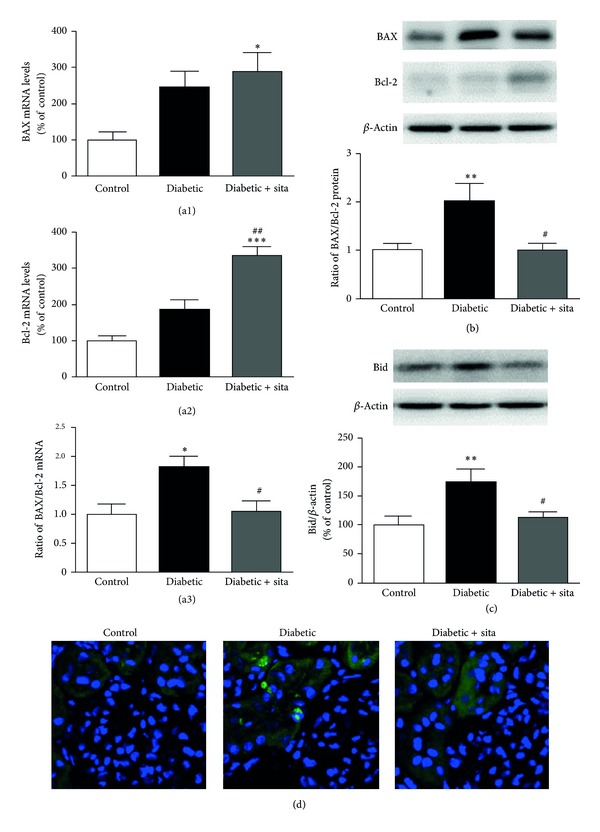
Effect of sitagliptin treatment in BAX, Bcl-2, and Bid content and TUNEL-positive cells in the diabetic kidney. (a) mRNA expression of BAX (a1) and Bcl-2 (a2) in the kidney and BAX/Bcl-2 ratio (a3). (b) The protein levels of BAX and Bcl-2 and BAX/Bcl-2 ratio were assessed in total kidney cell lysates by Western Blotting in ZDF (+/+) and ZDF (*fa/fa*) nontreated or treated with sitagliptin. (c) The protein levels of Bid were assessed in total kidney cell lysates by Western Blotting in ZDF (+/+) and ZDF (*fa/fa*) nontreated or treated with sitagliptin. The Western Blots presented are representative of each group of animals. Data are expressed as percentage of control and represent the mean ± SEM (*n* ≥ 6 per group); *P < 0.05, **P < 0.01, and ***P < 0.001 significantly different from control; ^#^ *P* < 0.05 and ^##^ *P* < 0.01 significantly different from diabetic. (d) Representative confocal images for each group of animals showing TUNEL-positive (green) cells and nuclear counterstaining with DAPI (blue) in kidney sections (6 *μ*m).

The diabetic animals presented significant increases in the kidney mRNA and protein of BAX/Bcl-2 ratio (Figures 6(a3) and 6(b), resp.), when compared to control rats. Sitagliptin treatment showed an antiapoptotic effect since it was able to prevent the diabetes-induced increment of mRNA (Figure 6(a)) and protein (Figure 6(b)) BAX/Bcl-2 ratio. Diabetes also induced a significant increase in Bid levels (173.57 ± 22.64% of control; *P* < 0.01) comparatively to nondiabetic ZDF (+/+) rats, which was prevented in diabetic rats under sitagliptin treatment (113.02 ± 9.43%; *P* < 0.05) (Figure 6(c)).

As shown in Figure 6(d), apoptosis increased in the kidney of diabetic animals, as assessed by the increase of TUNEL-positive cells, particularly in tubular cells. Treatment with sitagliptin decreased the number of TUNEL-positive cells in the kidney of diabetic animals (Figure 6(d)).

## 4. Discussion

In the present study, we showed that daily oral administration of sitagliptin for 6 weeks reduced glycaemia and HbA_1c_ (by about 1.2%) levels in diabetic ZDF rats. In addition, metabolic manipulation of DPP-IV inhibition to prevent degradation of GLP-1 can prevent the inflammatory processes and the proapoptotic state in the diabetic kidney, which might explain the amelioration of renal dysfunction and glomerular and tubulointerstitial damage. These findings demonstrate the beneficial effects of long-term sitagliptin administration in the kidney of this animal model of T2DM.

Several reports have suggested that sitagliptin, a DPP-IV inhibitor, confers cytoprotective effects on several tissues, such as heart, kidney, pancreas, and retina [32, 33, 35, 47, 48]. Our group has recently shown that sitagliptin corrected the glycaemic dysmetabolism, hypertriglyceridaemia, and inflammation, reduced the severity of histopathological pancreatic lesions, and prevented kidney lesions [35, 44]. However, until the present study, there was no evidence in the literature of whether the sitagliptin restores kidney function, in terms of improvement of inflammatory state and cell death, either by decreasing blood glucose levels or by extrapancreatic action of incretins in an independent manner of increased insulin secretion.

To determine the putative benefits of sitagliptin treatment in the diabetic kidney, and the underlined mechanisms, the ZDF rats were used as a T2DM model. Rodent models of T2DM are frequently used to clarify the mechanisms responsible for the pathophysiology of diabetes evolution, as well as its complications, such as DN. The disorder in human patients is characterized by renal hypertrophy, hyperfiltration, proteinuria, and progressive glomerulosclerosis [2]. Similar to patients with clinical diabetes, the ZDF rat develops diabetic complications, including nephropathy [40, 41]. Accordingly to previous studies, ZDF (*fa/fa*) rats show a significant renal hypertrophy at 20 weeks of age, as well as loss of kidney function [35]; thus, this animal model seems to be useful for evaluation of the effects of sitagliptin treatment in the diabetic kidney and their benefits in the prevention or reduction of some of the lesions present in DN.

Our results showed that chronic treatment with sitagliptin is able to promote a partial, but significant, decrease of glycaemia and HbA_1c_ and also prevented the aggravation of weight loss, characteristic of a more advanced stage of T2DM, which confirms the results obtained in studies performed in humans [24–28].

In an attempt to clarify whether sitagliptin improves the glycaemic control by inhibition of DPP-IV leading to an enhancement of the “incretin effect,” we evaluated the kidney DPP-IV protein levels. DPP-IV is widely distributed in numerous tissues and cells, and its enzymatic activity is exhibited in both membrane-anchored cell-surface peptidase and as a smaller soluble form in blood plasma [49]. In this study, we assessed DPP-IV protein levels in the kidney tissue, through the membrane form, also called CD26. We found that diabetes induced an increase in kidney DPP-IV protein levels, when compared to nondiabetic animals. Accordingly to our results, it was reported in a streptozotocin (STZ-) induced diabetic model that enhanced expression of* Dpp-IV* mRNA in the kidney tissue is correlated with increased circulating DPP-IV enzyme activity, although DPP-IV activity in the tissue was found to be significantly reduced after STZ treatment [50]. It may be reasonable that hyperglycemia-induced kidney cell damage may lead to enhanced biosynthesis of DPP-IV enzyme and its secretion by endothelial cells into the circulation, as suggested in previous reports [51, 52]. In fact, there are also some reports suggesting that microvascular endothelial cells are the main sources of endogenous DPP-IV [53] and* in vitro* studies showed that both* Dpp-IV* mRNA expression and DPP-IV activity were enhanced by exposure of human glomerular endothelial cells to high glucose [51]. Our results show that treatment with the DPP-IV inhibitor sitagliptin led to the accumulation of GLP-1, a DPP-IV substrate, in the diabetic kidney, which suggests that the content and/or activity of DPP-IV present in the kidney may be responsible for the observed changes in GLP-1 levels. In addition, we found reduced expression of GLP-1R in the diabetic rats, which was restored in the sitagliptin-treated rats, suggesting a role of GLP-1R signaling in the amelioration of nephropathy, according to other reports [54]. Furthermore, GLP-1R colocalizes with GLP-1 expression, suggesting that the renoprotective effects of sitagliptin may derive, at least in part, from GLP-1/GLP-1R activation. It was recently suggested that sitagliptin, via GLP-1 stabilization, is able to promote cardioprotection in a model of type 2 diabetic by reducing and limiting hyperglycemia and hyperlipidemia, as well as by promotion of survival and antihypertrophic/fibrotic effects on cultured cardiac cells, via GLP-1 and GLP-1(9-36), suggesting cell-autonomous cardioprotective actions [55]. In fact, although cytoprotective properties on renal tissue derived from the amelioration of insulin cannot be excluded, the results obtained in type 1 diabetes models [56, 57] suggest extra-pancreatic (renoprotective) effects of GLP-1/GLP-1R. Whether the kidney GLP-1 contents are derived from the amount produced by the gut, which may arise in the kidney via the circulation, or are the result of local production, they deserve further elucidation. We presented evidence of renal colocalization of GLP-1 and GLP-1R, thus suggesting the existence of sitagliptin effects other than those resulting from improved glycaemic/insulinemic control.

Accumulating evidences point to a critical role of inflammation and proinflammatory cytokines in the development and progression of DN [10, 11]. Our results clearly indicate that diabetes leads to increased IL-1*β* and TNF-*α* immunoreactivity in the kidney. These results are corroborated by other authors that described an increased expression of those proinflammatory cytokines in the diabetic kidney [58], leading to enhanced vascular endothelial permeability, oxidative stress, renal hypertrophy, and tubulointerstitial lesions [13]. Recently, it has been reported that in the kidney of ZDF (*fa/fa*) rats the expression of vascular cell adhesion molecule-1 increases with concomitant infiltration of white blood cells, as well as enhanced production of inflammatory cytokines, such as TNF-*α* and IL-1*β*, leading to renal cells injury [59].

Previous works have shown that the decrease in inflammation promotes an amelioration of diabetic nephropathy [35–37, 60]. In the present study we found that sitagliptin was able to prevent the increase in both mRNA and protein levels of the proinflammatory cytokines IL-1*β* and TNF-*α* in the diabetic kidneys. These results, obtained by immunohistochemistry and RT-qPCR in the kidney, seem to be in agreement with previous studies from our group, which report decreased IL-1*β* and TNF-*α* levels in the serum [44] and retina [31] of ZDF (*fa/fa*) rats treated with sitagliptin. Together, these findings seem to indicate that a chronic sitagliptin treatment corrected the inflammatory state in diabetic microvascular complications, such as DN. In addition, it was reported that sitagliptin decreases local inflammation in other tissues, such as the adipose tissue and pancreatic islet of obese mice [61].

Moreover, our immunohistochemistry studies also revealed that sitagliptin was able to prevent the diabetes-induced increase in IL-1*β* and TNF-*α* mainly in the cells around the glomeruli, which are probably tubular cells and/or accumulation of interstitial inflammatory cells. It has been shown that inflammatory cells, such as macrophages, lymphocytes, and monocytes, are often found in tubular compartment [62]. In fact, there are reports of infiltration of mononuclear cells in the kidney of patients with DN [63], showing that interstitial inflammatory cells infiltrates are associated with progression of renal injuries in DN [64, 65]. MCP-1 played a key role in promoting recruitment and infiltration of macrophage in the diabetic kidney [66], and it has been described that hyperglycemia increases expression of MCP-1 in tubular cells of the diabetic kidney [62, 66]. The proinflammatory transcription factor NF-*κ*B was also detected mainly in tubular cells of human and rat kidney, with T2DM and overt nephropathy [67]. Furthermore, the NF-*κ*B regulates the gene expression of several molecules involved in inflammation, which includes MCP-1, IL-1*β*, and TNF-*α* [68]. Based on these evidences, the NF-*κ*B and MCP-1 increased expression in tubular cells of diabetic kidney can be a plausible explanation for our results, but to clarify this hypothesis further studies are required.

The activation of signaling pathways linked to cell death resulting from chronic hyperglycemia and a state of low-grade chronic inflammation contributes to an increase in apoptosis. It is well established that members of the Bcl-2 family are key regulators of cell death. In the present study, a proapoptotic state seems to be favored in the kidney of diabetic ZDF rats, which can lead to loss of renal cells and consequent renal function loss [15, 16]. This increase in cell death by apoptosis appears to be mediated by BAX and Bid. Additionally, it has been demonstrated that glucose-induced ROS production initiates podocyte apoptosis and its depletion both* in vitro *and* in vivo*, leading to DN [17]. Therefore, a good glycaemic control could reduce the ROS production and the consequent risk of cell death. In addition, sitagliptin was able to ameliorate serum TGs contents, thus reducing lipotoxicity-evoked apoptosis in the kidney tissue [69].

Accordingly to what was previously reported in the retina of ZDF rats, treatment with sitagliptin reduced this proapoptotic state and cell death by apoptosis in the kidney [31]. It has been previously described that activation of incretin receptors in pancreatic *β*-cells promotes resistance to apoptosis through the activation of several pathways leading to inhibition of caspase-3, by increasing expression of Bcl-2 and decreasing expression of BAX [70, 71]. Moreover, GLP-1 has antiapoptotic actions via alteration of the Bcl-2 family proteins in several cell types. In fact, it was found that GLP-1 upregulates Bcl-2 and inhibits BAX expression in cholangiocytes [72], neuronal cells [73, 74], and endothelial cells [75]. In addition, it was shown that GLP-1 enhances Bcl-2 upregulation [76], BAD inactivation [77], and caspase-3 activity reduction [78] in pancreatic *β* cells. Here, we confirmed and expanded these data by demonstrating that increased levels of GLP-1 through DPP-IV inhibition prevents the BAX/Bcl2 mRNA and protein increase and reverses the increase in Bid and TUNEL-positive cells induced by chronic hyperglycemia in the ZDF kidney rats.

In summary, our results show that the improvement of renal lesions could be related to the sitagliptin-induced prevention of inflammation and apoptosis induced by diabetes. Consistent with this, other studies have demonstrated that GLP-1 receptor activation also attenuated diabetic renal injury, including reduction of renal oxidative stress [79] and suppression of renal inflammatory cytokines [56]. Therefore, the anti-inflammatory and antiapoptotic effect of sitagliptin can be related to direct activation of GLP-1 receptor, highlighting the importance of sitagliptin as a therapeutic agent for diabetic nephropathy.

## 5. Conclusions

In the present study, we confirm the deleterious effects of diabetes in the kidney, resulting in cell death by apoptosis and increased inflammation and that these are accompanied by increased DPP-IV protein levels and a reduction in GLP-1 protein levels. Sitagliptin treatment restored DPP-IV and GLP-1 levels in the diabetic kidney towards those in the control group. Furthermore, sitagliptin was able to ameliorate nephropathy induced by diabetes in a T2DM animal model, the ZDF (*fa/fa*) rats, due to anti-inflammatory and antiapoptotic properties. In conclusion, sitagliptin might be viewed as a promising preventive renoprotective therapeutic strategy against the development and/or progression of diabetic nephropathy.

## Figures and Tables

**Table 1:** Body weight and glycaemia in the control and diabetic ZDF rats, nontreated and treated with sitagliptin for 6 weeks.

Time	Initial time (20 wks)		Final time (26 wks)		
Rat group	Control	Diabetic	Control	Diabetic	
Parameters	(*n* = 8)	(*n* = 16)	Vehicle (*n* = 8)	Vehicle (*n* = 8)	Sita (*n* = 8)
BW (g)	407.8 ± 7.3	394.6 ± 6.5	438.2 ± 4	370.4 ± 11.8**	389.5 ± 15.7
Glycaemia (mg/dL)	92.3 ± 2.5	304.6 ± 9.1***	88.8 ± 2.9	425.4 ± 10.3***	329.8 ± 24.4^###^

Values are means ± SEM of *n* rats. **P < 0.01 and ***P < 0.001 significantly different from age-matched control; ^###^ *P* < 0.001 significantly different from age-matched diabetic.
